# The Effects of Different Probiotic Administration on Dexamethasone-Associated Metabolic Effects

**DOI:** 10.3390/microorganisms13040739

**Published:** 2025-03-25

**Authors:** Andreea Ioana Inceu, Maria Adriana Neag, Corina Ioana Bocsan, Anca Elena Craciun, Carmen Stanca Melincovici, Dana Maria Muntean, Florentina Claudia Militaru, Mădălin Mihai Onofrei, Raluca Maria Pop, Luciana-Mădălina Gherman, Marius Bichescu, Anca Dana Buzoianu

**Affiliations:** 1 Department of Morpho-Functional Sciences, Discipline of Pharmacology, Toxicology and Clinical Pharmacology, “Iuliu Hatieganu” University of Medicine and Pharmacy, 400012 Cluj-Napoca, Romania; 2 Heart Institute “Niculae Stancioiu” Cluj-Napoca, 400001 Cluj-Napoca, Romania; 3 Department of Medical Specialties, Discipline of Diabetes and Nutrition Diseases, “Iuliu Hatieganu” University of Medicine and Pharmacy, 400012 Cluj-Napoca, Romania; 4 Department of Morpho-Functional Sciences, Discipline of Histology, “Iuliu Hatieganu” University of Medicine and Pharmacy, 400012 Cluj-Napoca, Romania; 5 Discipline of Pharmaceutical Technology and Biopharmaceutics, “Iuliu Hatieganu” University of Medicine and Pharmacy, 400012 Cluj-Napoca, Romania; 6 Experimental Centre, “Iuliu Hatieganu” University of Medicine and Pharmacy, Louis Pasteur Street No. 6, 400349 Cluj-Napoca, Romania

**Keywords:** dexamethasone, hyperglycemia, hypercholesterolemia, hepatic steatosis, probiotics, inflammation, oxidative stress

## Abstract

Glucocorticoids are steroid hormones used in clinical practice as an effective therapeutic option for their effects regarding the dysregulated immune reactions and hyperactive immune system. Their administration in the short- and long-term exposure has been associated with numerous metabolic side effects. Probiotics have been shown to modulate basal metabolism, inflammation, and oxidative stress through the regulation of composition and function of the gut microbial environment. The aim of this study was to assess the effects of *Saccharomyces boulardii* and *Lactobacillus paracasei* probiotics in dexamethasone-treated rats. The study comprised four groups, with 6 Charles River Wistar albino male rats/group; group 1 represented the negative control, rats from group 2 were administered dexamethasone, rats from group 3 were administered dexamethasone and probiotics containing the strain *Saccharomyces boulardii*, and rats from group 4 were administered dexamethasone and probiotics containing the strain *Lactobacillus paracasei*. We have assessed the plasmatic levels of glucose, total cholesterol, triglycerides, tumor necrosis factor-alpha, interleukin-10, catalase activity, and total antioxidant capacity. The administration of dexamethasone led to elevated serum concentrations of glycolipid metabolism parameters and cytokines and resulted in hepatic steatosis at the morphological level. Administration of probiotics containing *Saccharomyces boulardii* or *Lactobacillus paracasei* reduced glucose and tumor necrosis factor-alpha serum concentration in dexamethasone-treated rats. Moreover, the administration of *Lactobacillus paracasei* probiotics in rats that received dexamethasone increased interleukin-10 and reduced catalase activity. Regarding the liver tissue morphology, the rats that received probiotics showed improved liver histological aspects compared to the dexamethasone-treated group, suggesting that probiotics could provide positive effects regarding the metabolic and histological disturbances induced by glucocorticoids.

## 1. Introduction

Glucocorticoids are one of the well-established alternatives for the treatment of inflammatory disorders, due to their effective properties against dysregulated inflammatory response. Chronic glucocorticoid administration promotes the development of adverse effects regarding basal metabolism, such as insulin resistance, glycemia disturbances, dyslipidemia, central adiposity, and hepatic steatosis, leading over time to the development of steroid diabetes, a type of diabetes mellitus [1]. During oral glucocorticoid therapy, the development of new-onset diabetes mellitus was approximated to 2% of the primary care population [2]. A meta-analysis revealed that in non-diabetic patients using glucocorticoids for longer than a month, the incidence of diabetes mellitus was 19% and the incidence of glucocorticoid-induced hyperglycemia was 32% [3]. For hospitalized patients, glucocorticoid-induced hyperglycemia increases the risk of mortality and morbidity, leading to a higher risk for infectious diseases and major cardiovascular events. The treatment of glucocorticoid-induced hyperglycemia relies on insulin administration depending on the type and duration of action of glucocorticoid therapy [4]. Dexamethasone (Dexa) is a long-acting glucocorticoid that has been associated with the development of metabolic disorders, such as insulin resistance and non-alcoholic fatty liver disease (NAFLD), in animal studies [5,6]. Moreover, Dexa administration induced morphological and functional alterations within the colon with increased local inflammatory infiltration and the gut barrier regarding microbiota richness and composition [7].

Probiotics are defined as “live microorganisms, which when administered in adequate amounts, confer a health benefit on the host”, as reinforced in the International Scientific Association for Probiotics and Prebiotics consensus [8]. The administration of probiotics in the form of wide-type probiotics, combined probiotics, or recombinant probiotics acts in the onsetting and development of diabetes mellitus type 2 by several pathways, such as modulation of energy metabolism, gut microbiota composition and function, and local intestinal immune system. Their use has been associated with improved metabolic and inflammatory status, direct antidiabetic properties, and amelioration of the gut barrier structure and function, reducing systemic bacterial translocation [9]. In both animal studies with experimentally induced diabetes by administration of a personalized diet or specific drugs and patients diagnosed with diabetes mellitus type 2, the administration of probiotics, mainly containing *Lactobacillus* strains, was associated with attenuation of the insulin resistance state and improved glucose metabolism and modulation of gut microbiota structure and function. The proposed molecular mechanisms were related to the increased abundance of short-chain fatty acids (SCFA)-producing bacteria and the inhibition of pathogenic microorganisms [10]. For example, *Lactobacillus paracasei* (LP) administration, particularly the HII01 strain, has shown valuable effects regarding glycemic metabolism, improving the insulin-stimulated glucose uptake and the systemic metabolic profile by reducing the plasmatic levels of glucose, lipids, and the hepatic deposition of triglycerides in an animal model of experimentally induced diabetes. Moreover, the LP HII01 strain was involved in regulating the gut microbiota composition and function by restoring intestinal permeability, reducing systemic endotoxemia and inflammation levels, and modulating the SCFA generation [11]. Another LP strain, the L14 strain, showed similar effects regarding dyslipidemia, inflammation, and glucose-related function of pancreatic β-cells, while supplementary effects in the decrease in oxidative stress parameters were described; LP L14 strain contains 16 antioxidant-related genes, such as the ones belonging to the thioredoxin system and the glutathione–glutaredoxin system; it also has been associated with the biosynthesis of exopolysaccharides, providing evidence for particular antioxidant effects [12]. In different studies of type 2 diabetes mellitus rat models, probiotics containing various strains of LP, such as the NL41 strain or NTU 101, promoted the regulation of glucose and lipid metabolism by improving plasmatic and histological markers and modulating the function of pancreatic and intestinal cells [13,14]. On the other hand, in streptozotocin-induced mice, probiotics containing *Saccharomyces boulardii* (SB) THT 500101 strain were associated with reduced plasmatic levels of glucose and triglyceride, increased peptide C levels, and hepatic accumulation of glycogen; furthermore, the same SB strain regulated the inflammation profile and gut microbiota composition, suggesting multiple systemic effects responsible for metabolic actions [15].

There is scarce information about the role of probiotic administration in dexamethasone-induced metabolic disturbances. We have previously shown that the administration of a probiotic based on *Bacillus* spores could ameliorate the lipid dysregulation induced by dexamethasone [16]. However, there are no published studies regarding the effects of other probiotic supplements in glucocorticoid-treated rats.

Therefore, this study aimed to assess the effects of two different probiotic supplements containing two of the most-studied bacterial species in metabolic disorders, SB or LP in Charles River Wistar albino rats previously treated with dexamethasone, regarding the metabolic and inflammation profile, oxidative stress parameters, and liver tissue morphology.

## 2. Materials and Methods

### 2.1. Agents and Chemicals

In the current study, the used agents were Dexamethasone Sodium Phosphate (Dexa) Krka 4 mg/mL (KRKA d.d. Novo mesto, Novo mesto, Slovenia), probiotic strains *Saccharomyces boulardii* (SB) lyo CNCM I-745 comprised in a product from BIOCODEX (Gentilly, France) and *Lactobacillus paracasei* (LP) CNCM I-1572 comprised in a product from SOFAR (Bucharest, Romania). Every 250 mg of SB probiotic product contains five billion colony-forming units (CFU) of SB lyo CNCM I-745. The LP product contains 24 billion CFU of LP CNCM I-1572. All products were purchased from a public pharmacy. For the administration of probiotics, the oral route was used; the volume of administration was 1 mL suspension of 1% carboxymethylcellulose (CMC), as vehicle-Sigma-Aldrich (Taufkirchen, Germany). Dexa was administered using the intraperitoneal (i.p.) route.

### 2.2. Animals

The animals were acquired from the Center for Experimental Medicine and Practical Skills of Iuliu Hatieganu University of Medicine and Pharmacy from Cluj-Napoca. 24 Charles River Wistar albino male rats, weighing 230 and 280 g, were used in this current study, with housing and handling conditions similar to our previous work [16]. The Ethics Committee of Iuliu Hatieganu University of Medicine and Pharmacy (no. AVZ262/15.09.2022) and the National Sanitary Veterinary and Food Safety Authority (no. 336/14.10.2022) revised and approved the protocol for this experimental study. Specific regulations from the “Guiding Principles in the Use of Animals in Toxicology” adopted by the Society of Toxicology (Reston, VA, USA) and the specific national laws were applied for scientific research, taking into consideration the protection of the animals. The rats were under general anesthesia for blood collection and other invasive maneuvers, and on the final day of the study, the animals were sacrificed with an overdose of xylazine/ketamine, all efforts being made to minimize suffering.

### 2.3. Experimental Design

The study groups contained 6 rats in each study group after the randomization of the 24 Wistar male rats into 4 groups. The study duration was 7 days for chemical compound administration and was similar to our previous research [16]. Group 1 served as the negative control group: 1% CMC as the vehicle was administered orally, and saline solution was administered i.p.; group 2 (Dexa) served as the positive control group: Dexa 1 mg/kg bw/day was administered i.p. and the vehicle, 1% CMC, orally; rats from group 3 (Dexa+ SB) were administered probiotics containing SB orally (2.5 × 10^9^ CFU/day/animal); rats from group 4 (Dexa+ LP) were administered probiotics containing LP orally (3 × 10^8^ CFU/day/animal); additionally, rats from groups 3 and 4 received i.p. Dexa 1 mg/kg bw/day. The blood was collected before feeding and was processed as shown in our previous work [16]. The experimental design is shown in Table 1.

### 2.4. Evaluation of Inflammatory and Biochemical Markers

The inflammatory markers assessment, which included tumor necrosis factor-alpha (TNF-α) and interleukin-10 (IL-10), was performed using the enzyme-linked immunosorbent assay (ELISA) method, with ELISA-specific kits (Rat TNF-α Standard TMB ELISA Development Kit; PeproTech Inc., Rocky Hill, NJ, USA; Rat IL-10 ELISA Kit; Elabscience, Houston, Texas, USA as in our previous studies [16].

### 2.5. Assessment of Oxidative Stress Parameters

Total antioxidant capacity (TAC) was measured through a method previously described by Erel [17]. This technique was validated and displayed in other similar studies and focuses on the antioxidant’s ability to interfere with 2,2′-azinobis-(3-ethylbenzothiazoline-6-sulfonate (ABTS^+^) [16,18]. Trolox, a water-soluble analog of vitamin E, was utilized for the calibration curve; the TAC results were reported as mmol Trolox equivalent/L.

Catalase activity was measured by using a method based on a UV spectrophotometer (Specord 250 Plus, Analytik Jena, Jena, Germany), as previously explained by Aebi [19]. This method was described in other similar studies conducted by our lab [16,18].

### 2.6. Histological Analysis

The removed liver tissue samples were processed using the methods previously described [16].

Histological analysis of liver samples assessed the following variables: alterations of normal hepatic lobule architecture, morphological changes in hepatocytes, intrahepatocyte lipid accumulation, intralobular or portal inflammation, and congestion within the vascular system of the liver. According to the Brunt system [20,21], to calculate the Non-alcoholic Fatty Liver Disease Activity Score (NAS), we have assessed several main hepatic lesions in our groups: steatosis (0–3), inflammation within the hepatic lobule (0–3), inflammation within the portal space (0–2), hepatocellular ballooning (0–2), and fibrosis (0–3). The grading and staging system for non-alcoholic steatohepatitis was classified in grade 1 (mild), grade 2 (moderate), and grade 3 (severe).

### 2.7. Statistical Analyses

The obtained results were reported as mean ± standard deviation. Firstly, the distribution of the data was assessed through the Shapiro-Wilk test and QQ plot representation. Once the normal distribution was checked, a one-way analysis of variance was used for the comparison between different groups. The post-hoc Tukey correction was then performed to compare the pairs of groups. Statistical significance was set for a *p*-value less than 0.05. The statistical tests were completed using GraphPad Prism, version 10.4.1. (GraphPad Software, Boston, MA, USA).

## 3. Results

### 3.1. Metabolic Parameters

**Figure 1:**
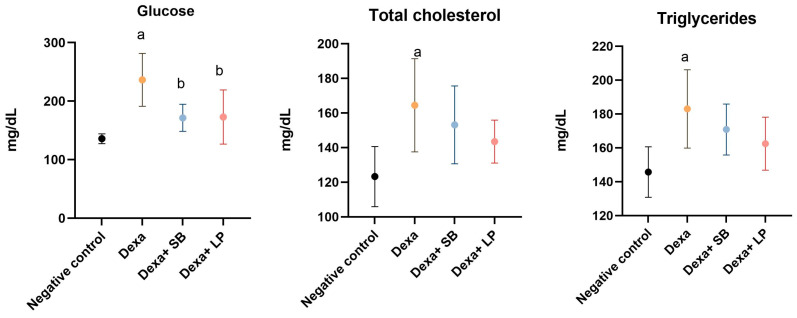
Serum metabolic biomarkers concentration. a: *p* < 0.05 compared to the negative control group; b: *p* < 0.05 compared to the Dexa group; Abbreviations: Dexa, dexamethasone; SB, *Saccharomyces boulardii*; LP, *Lactobacillus paracasei*. The lines correspond to mean values with standard deviation.

The serum concentrations of glucose, total cholesterol, and triglycerides are represented in Figure 1.

Glucose serum concentration was increased significantly in the Dexa group versus the negative control group (236.4 ± 45.3 mg/dL versus 135.8 ± 8.38 mg/dL, respectively, *p* < 0.05). The administration of SB probiotics or LP probiotics in Dexa-treated rats decreased significantly the glucose in the serum compared to the Dexa group (171.5 ± 23.06 mg/dL, *p* < 0.05, and 172.8 ± 46.27 mg/dL, *p* < 0.05, respectively). The glucose serum concentration was assessed before feeding.

In the negative control group, the concentration of total cholesterol was 123.3 ± 17.37 mg/dL. Dexa administration increased significantly total cholesterol (164.5 ± 26.94 mg/dL, *p* < 0.05) versus the negative control group. In rats that received both Dexa and SB or LP probiotics, total cholesterol decreased by 7% and 12.7%, respectively, compared to the Dexa group, but the differences did not reach the *p*-value for statistical significance (*p* = 0.77 and *p* = 0.31, respectively).

Triglyceride serum levels were significantly increased in the Dexa group (183 ± 23.14 mg/dL, *p* < 0.05) compared to the negative control group (145.7 ± 14.89 mg/dL). In rats that received Dexa and SB or LP probiotics, triglyceride serum concentration decreased by 6.77% and 11.2%, respectively, but without a statistically significant level, compared to the Dexa group.

### 3.2. Inflammation Cytokines

**Figure 2:**
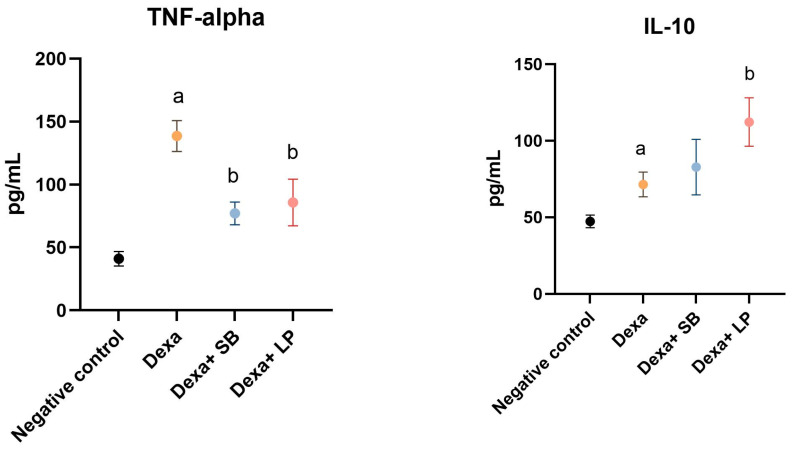
Serum inflammation cytokines levels. a: *p* < 0.05 compared to the negative control group; b: *p* < 0.05 compared to the Dexa group. Abbreviations: TNF-α, tumor necrosis factor alpha; IL, interleukin; Dexa, dexamethasone; SB, *Saccharomyces boulardii*; LP, *Lactobacillus paracasei*. The lines correspond to mean values with standard deviation.

The serum levels of TNF-α and IL-10 are shown in Figure 2.

Dexa administration caused a significant elevation of TNF-α in the serum of Dexa-treated rats versus the negative control group (138.5 ± 12.26 pg/mL versus 41.06 ± 5.73 pg/mL, *p* < 0.05). Administration of SB probiotics in Dexa-treated rats significantly reduced the concentration of TNF-α (77.15 ± 8.98 pg/mL, *p* < 0.05) versus the Dexa group. LP probiotics administration in Dexa-treated rats also significantly decreased the concentration of TNF-α (85.73 ± 18.51 pg/mL, *p* < 0.05).

In the Dexa-treated group, the IL-10 serum levels were significantly elevated (71.49 ± 8.07 pg/mL, *p* < 0.05) versus the Control group (47.29 ± 4.13 pg/mL). SB probiotics administration in Dexa-treated rats increased the IL-10 levels (82.77 ± 18.12 pg/mL) versus the Dexa group by 15.7%, but the results were not statistically significant. In rats that received LP probiotics and Dexa, IL-10 values were significantly higher versus the Dexa-treated rats (112.2 ± 15.81 pg/mL, *p* < 0.05).

### 3.3. Oxidative Stress Parameters

**Figure 3:**
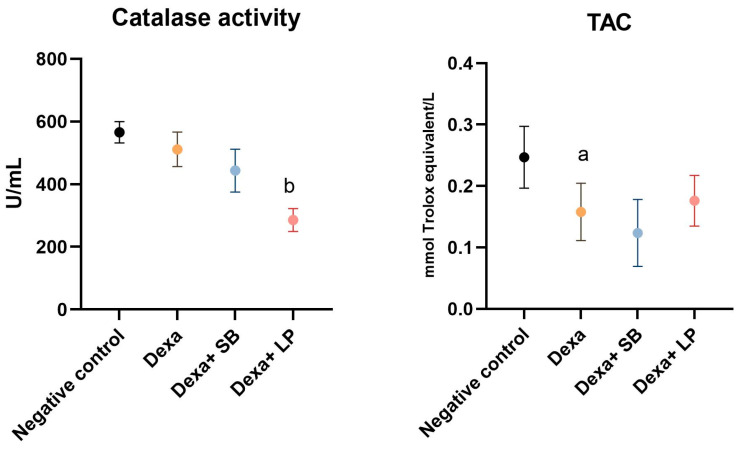
Catalase activity and TAC levels. a: *p* < 0.05 compared to the negative control group; b: *p* < 0.05 compared to the Dexa group. Abbreviations: TAC, total antioxidant capacity; Dexa, dexamethasone; SB, *Saccharomyces boulardii*; LP, *Lactobacillus paracase*. The lines correspond to mean values with standard deviation.

Catalase activity and TAC are shown in Figure 3.

Catalase activity was decreased in the Dexa group (511.3 ± 55.02 U/mL) compared to the negative control group (565.7 ± 33.76 U/mL), but without statistically significant results. In the Dexa+ SB probiotics rats, catalase activity did not show statistically significant differences (*p* = 0.12) compared to the Dexa group. However, administration of LP probiotics in Dexa-treated rats significantly reduced catalase activity (285.7 ± 36.70 U/mL, *p* < 0.05) versus Dexa-treated rats.

TAC significantly decreased in the Dexa group (0.157 ± 0.04) compared to the negative control group (0.246 ± 0.05). The administration of SB or LP probiotics in Dexa-treated rats did not alter significantly the TAC compared to the Dexa group, although the Dexa and LP probiotics group exhibited elevated TAC (0.176 ± 0.04, *p* = 0.91).

### 3.4. Histological Examination Results

**Figure 4:**
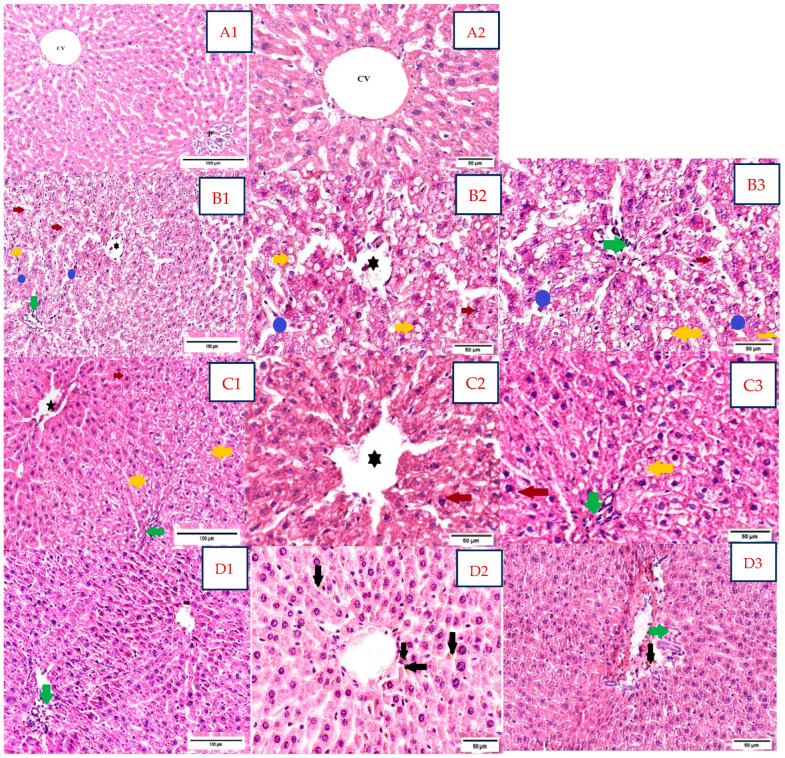
Rat liver sections in the study groups stained with H&E (**A1**,**A2**,**B1**–**B3**,**C1**–**C3**,**D1**–**D3**). Abbreviations: H&E, hematoxylin, and eosin. (**A**). Negative control group; H&E stain; (**A1** 20×) and (**A2** 40×). Normal disposition of hepatocytes within the hepatic lobule, with normal thickness of hepatic cords arranged around the central vein (CV); normal aspect of the portal space (P). (**B**). Dexa-treated rat group; H&E stain; (**B1** 20×) and (**B2** 40×, **B3** 40×). Ballooning degeneration of hepatocytes with small lipid droplets in the perinuclear area (red arrow); macrovesicular steatosis (yellow arrow); vascular congestion and inflammatory infiltrate in the central vein (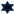); moderately dilated sinusoids with mild inflammatory infiltrate (polymorphonuclears neutrophils) within the sinusoids (blue circle); portal mixed inflammatory infiltrate (green arrow); NAS score moderate, grade 2: steatosis—grade 3; ballooning degeneration—grade 2; lobular inflammation—grade 1 (<2 foci/×20 field), portal inflammation—grade 1. (**C1**–**C3**). Dexa+ SB probiotics group; H&E stain; (**C1** 20×) and (**C2** 40×, **C3** 40×). Restoration of lobular architecture, with a marked reduction in macrovesicular steatosis (yellow arrow); ballooning degeneration of hepatocytes with small lipid droplets in the perinuclear area (red arrow); the portal triads exhibited moderate lymphocytic inflammatory infiltrate (green arrow); stasis of central vein (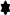); NAS score mild to moderate, grade 1/2: steatosis—grade 1, ballooning degeneration—grade 2, lobular inflammation—grade 2 (2–4 foci/×20 field), portal inflammation—grade 2. (**D1**–**D3**). Dexa+ LP probiotics group; H&E stain; (**D1** 20×) and (**D2** 40×, **D3** 40×). Normal lobular architecture, with the restoration of hepatocyte shape and structure; focal lymphocytic inflammatory infiltrate in the portal triads was maintained (green arrow); focal hemosiderin pigment (black arrow) was spotted in the centrilobular zone and the connective tissue belonging to the portal triads; NAS score mild, grade 1: steatosis—grade 1, ballooning—grade 1, lobular inflammation—grade 0, portal inflammation—grade 1.

Histological analysis of liver samples assessed the following variables: alterations of normal hepatic lobular architecture, morphological changes in hepatocytes, intrahepatocyte lipid accumulation, intralobular or portal inflammation, and congestion within the vascular hepatic system (Figure 4).

The negative control group (Figure 4(A1,A2)) exhibited a normal distribution of the hepatocyte cords and adjacent sinusoids following the central hepatic vein, displaying a regular architecture within the hepatic lobules.

Dexa-treated rats showed alterations of the hepatic lobules architecture, with a moderate NAS score, grade 2 (Figure 4(B1–B3)). The main structural characteristics in this group were ballooning degeneration and intrahepatocyte lipid accumulation with macrovesicular steatosis. Large, rounded hepatocytes with edematous, pale eosinophilic cytoplasm were evident in the majority of the fragments, with small lipid droplets in the perinuclear area. Macrovesicular steatosis, mainly seen in the periportal areas (acinar zone 1), with extension in the acinar zone 2, was identified in approximately 70% of hepatocytes. Vascular congestion in the central vein with a few polymorphonuclear neutrophils within the lumen, as well as sinusoidal dilatation with mild inflammatory infiltrate, were identified. Portal triads displayed a mild, mixed inflammation with lymphocytes, plasma cells, and a few polymorphonuclear neutrophils.

Sections belonging to Dexa and SB probiotics rats (Figure 4(C1–C3)) exhibited mild restoration of lobular architecture, with a mild to moderate NAS score, grade 1/2. The liver samples of this group showed a marked reduction in steatosis with minimal macrovesicular steatosis in below 5% hepatocytes and small lipid droplets. Ballooning degeneration, with large hepatocytes with edematous, pale eosinophilic cytoplasm, was maintained. The portal triads and the centrilobular areas displayed focal, moderate lymphocytic inflammatory infiltrate. Meanwhile, scattered stasis of central veins was detected.

In rats treated with Dexa and LP probiotics (Figure 4(D1–D3)), we observed a quasi-normal lobular architecture, with a mild NAS score of grade 1. Restoration of hepatocyte shape and cytoplasmic aspect, with few intracellular lipid droplets and few features of ballooning degeneration, was described. The focal lymphocytic inflammatory infiltrate in the portal triads was maintained but in a reduced quantity compared to the previous groups. Focal hemosiderin pigment was spotted in the centrilobular zone.

We did not encounter any evidence of fibrosis in the usual H&E stain in any group.

## 4. Discussion

The use of glucocorticoids in acute and chronic inflammatory diseases due to their effective action against dysregulated inflammatory reactions is hampered by undesirable metabolic side effects, including the development of diabetes mellitus type 2 [1]. In this animal model of Dexa-induced metabolic disorders, a long-acting glucocorticoid, we assessed the effects of two probiotics containing different bacterial strains. SB and LP probiotic administration resulted in reduced metabolic markers, modulated inflammation profile, and decreased the hepatic storage of lipids in Dexa-treated rats. The observed effects are different compared to those previously reported by our lab in an animal model of glucocorticoid-induced metabolic effects, showing significant results over the metabolic and inflammatory parameters; administration of MSB reduced the serum total cholesterol and the hepatic lipid storage in Dexa-treated rats [16]. This current study shows the effects of different probiotics with different bacterial strains in Dexa-treated animals. The administration of SB probiotics reduced the serum glucose and the TNF-α levels, while the intake of LP probiotics decreased the glucose, the TNF-α concentration, and catalase activity and increased IL-10 levels; the histological alterations induced by Dexa were attenuated in both groups treated with SB and LP probiotics, with major improvement observed in the LP group. These results offer a more comprehensive analysis regarding the metabolic effects of different probiotic administration in Dexa-treated rats.

Dexa administration significantly altered the glycolipid profile by increasing serum glucose, total cholesterol, and triglycerides. These effects correspond to the ones reported in animal studies of Dexa-induced hyperglycemia and dyslipidemia [5,20]. Steroid-induced diabetes is among the well-established metabolic side effects of glucocorticoids and is associated with hyperglycemia, insulin resistance, dyslipidemia, central adiposity, and hepatic steatosis [1]. Although the onset of diabetes mellitus occurs predominantly with the long-term use of glucocorticoids, short-term treatment has also been associated with impaired insulin sensitivity and hyperglycemia in healthy individuals [21,22]. The actions of glucocorticoids are started by the interaction with their specific receptors in the cytoplasm of the target cell; the complex between the glucocorticoids and receptors then binds to the genome via glucocorticoid-response elements and modulates the gene expression. A multi-organ crosstalk is responsible for the metabolic side effects. Glucocorticoids promote leptin resistance and increase appetite in the brain; activate gluconeogenesis and the uptake of non-esterified fatty acid by hepatic cells, stimulating triglyceride production in the liver; decrease glucose use in adipocytes, enhance lipogenesis and triglyceride production, and facilitate lipolysis in an ineffective lipid cycle in the adipose tissue; promote proteolysis; and inhibit the glucose uptake in skeletal myocytes [1]. Moreover, Dexa administration in rats was associated with dysregulation of the microbiota composition regarding the bacterial phylum and family, colonic inflammatory cell accumulation, and structural alteration of the gut barrier by decreasing mucus secretion [7].

In the current study, probiotics with SB or LP significantly decreased serum glucose concentration compared to the Dexa-treated rats and reduced serum total cholesterol and triglycerides, but the differences in the lipid metabolism parameters were not statistically significant. Probiotics containing bacteria from the genera *Lactococcus*, *Lactobacillus*, *Bifidobacterium*, or *Saccharomyces* showed similar results regarding the metabolic biomarkers in experimental studies performed on animals with diet- or drug-induced diabetes and in patients diagnosed with diabetes mellitus type 2 [9]. The use of probiotics in preclinical and clinical settings displayed positive results regarding the development and the complications of diabetes mellitus type 2; the administration of probiotics through multiple mechanisms results in the improvement of gut microbiota composition and gut barrier function, regulation of basal energy metabolism through both direct action and secretion of bioactive molecules that are released into circulation, and modulation of the immune response, leading to ameliorated values of the metabolic and inflammation biomarkers [9,23]. A meta-analysis of forty-six randomized controlled trials showed that probiotics and synbiotics administration reduced fasting plasma glucose, hemoglobin A1c, insulin serum levels, and insulin resistance in patients with prediabetes and type 2 diabetes mellitus [24]. The administration of the LP HII01 strain in streptozotocin-induced diabetes in adult male Wistar rats resulted in improved glycemic and lipid parameters and insulin-stimulated glucose uptake, together with modulation of gut microbiota composition and reduction of endotoxemia [11]. Moreover, SB probiotics administration in leptin-resistant obese and type 2 diabetic mice (*db*/*db*) was associated with reduced body weight and accumulation of fatty tissue, decreased liver steatosis, and hepatic and systemic concentration of inflammatory cytokines; colonic local effects were observed after SB treatment regarding the amelioration of the cecum morphological structure and gut microbiota composition [25].

Regarding the inflammation profile, Dexa administration led to significantly elevated levels of TNF-α and IL-10 versus the negative control group. Previous studies of Dexa-induced metabolic disturbances showed similar results regarding the inflammation markers and could be related to the progression of an insulin resistance state [20,26]. Although glucocorticoids are known to reduce inflammation in conditions of hyperactive immune reactions, they have been associated with up-regulation of the gene expression of pro-inflammatory cytokines, such as TNF-α and IL-6; the effects are related to the administration period and quantity [27]. TNF-α, secreted from adipocytes in insulin-resistance states [28], is involved in reduced insulin sensitivity by down-regulating the expression of glucose transporter type 4 and consecutively decreasing the glucose uptake in the adipose tissue [29] and altering the insulin receptor activity [30]. The pro-inflammatory state induced by glucocorticoids was associated with alterations of the gut microbial environment and the development of metabolic disorders, suggesting additional mechanisms responsible for the metabolic side effects of glucocorticoids [7]. IL-10 is produced by various immune cells and acts primarily as an anti-inflammatory molecule that counterbalances the secretion and effects of pro-inflammatory molecules, such as TNF-α, through a direct action of monocytes-macrophages [31]. In the current paper, Dexa administration significantly increased the serum levels of IL-10 compared to the negative control group. The effects of Dexa over IL-10 production were reportedly dose-dependent in a whole-blood cell culture treated with lipopolysaccharides (LPS), by increasing IL-10 secretion at reduced doses and decreasing it at high doses [32]. Thus, the increased IL-10 secretion in the current study could suggest an anti-inflammatory action for the pro-inflammatory state induced by Dexa administration.

The administration of SB or LP probiotics in rats treated with Dexa resulted in significantly lower levels of TNF-α and increased levels of IL-10 in the rats treated with Dexa, although the results of IL-10 concentration reached the level of statistical significance only for the probiotics containing LP. SB probiotics administration showed favorable results by decreasing the serum lipids and inflammation molecules associated with changes at the phylum, family, and genus levels of the gut microbial environment in leptin-resistant obese and type 2 diabetic mice; the modulation of gut microbiota composition was correlated with metabolic parameters [25,33]. In high-fat diet/low-dose streptozotocin-induced diabetic rats, the administration of LP strain L14 exerted positive properties by reducing hyperglycemia, dyslipidemia, and inflammation; it also showed beneficial effects regarding oxidative stress and gut microbiota composition and improved pancreatic and liver function. In the same study, the supplementation with LP strain L14 decreased the serum levels of TNF-α and increased the concentration of IL-10, suggesting that LP action of attenuating the biomarkers of insulin resistance is associated with the modulation of inflammatory balance and local gut microbiota effects [12]. Probiotics containing *Lactobacillus fermentum* MCC2759 and MCC2760 showed a similar tendency to upregulate the gene expression of IL-10 and downregulate the gene expression of pro-inflammatory molecules, such as TNF-α, IL-6, and IL-1β in an animal model of diabetes induced by the feeding of a personalized diet and administration of streptozotocin [34]. The attenuation of metabolic disturbances induced by Dexa in rats that were treated with probiotics containing SB or LP by decreasing serum levels of glucose and lipids together with modulation of the inflammation profile proposes multiple interactions between the intestinal, liver, and pancreatic environments by regulating multiple metabolic and inflammatory pathways. Rats that were administered probiotics containing LP exhibited significantly reduced serum glucose and TNF-α and elevated levels of IL-10, suggesting that the improvement within the glucose metabolism is associated with the regulation of systemic inflammation.

Reactive species, derived from normal cellular metabolic reactions, can chemically interact with cellular biomolecules, such as proteins, nucleic acids, and lipids, and consequently interfere with their composition and metabolic activity. However, there are several antioxidant mechanisms within the cells that aim to neutralize the effects of reactive species. Catalase, among the main cellular antioxidant enzymes, protects against cellular oxidative stress by breaking down the molecule of two hydrogen peroxides into oxygen and water. The impairment of the antioxidant balance in pancreatic β cells with reduced catalase expression and increased concentration of hydrogen peroxide has been associated with reduced insulin production, insulin resistance, and the development of type 2 diabetes mellitus. Decreased antioxidant system performance and consecutive excessive reactive oxygen species lead to lipid peroxidation and protein oxidation, producing β cell dysfunction and apoptosis, altered mitochondrial functionality, and progression of diabetes mellitus [35,36,37].

In the current research, the catalase activity was decreased in the group that received Dexa, although the results were not statistically significant. This result follows the significant decrease in TAC in Dexa-treated rats and is similar to that observed in other studies of steroid administration, suggesting that the metabolic alterations provoked by the glucocorticoid administration in the form of Dexa could be associated with reduced antioxidant activity and consecutive excessive production of reactive species [38,39,40,41]. Treatment with SB probiotics in Dexa-treated animals did not alter significantly the antioxidant system of catalase or total antioxidant capacity compared to the Dexa group. In a study of streptozotocin-induced diabetes in an animal model, the administration of SB THT 500101 strain probiotics for 8 weeks showed a tendency to recover superoxide dismutase and glutathione peroxidase activity [42]. The administration of probiotics containing LP in Dexa-treated rats produced a significant decrease in catalase compared to the Dexa group. Regarding catalase activity, there are contradictory results in the literature. Reduced catalase activity was observed in a double-blind placebo-controlled clinical trial of patients with rheumatoid arthritis who received *Lactobacillus casei* 01 probiotics [43]; however, different LP strains showed antioxidant properties in both in vitro and in vivo studies, suggesting that the obtained results could be due to the short administration of the probiotics; LP CCFM1223 administration in an animal model of LPS-induced liver injury resulted in improved inflammatory status and antioxidative enzyme activity, such as TAC, catalase, superoxide dismutase, and glutathione peroxidase, together with modulation of gut microbiota composition [44,45]. Moreover, LP-treated rats showed elevated TAC compared to the Dexa group, but without statistically significant differences. This observation indicates that LP probiotics could impact the antioxidant system by supplementary mechanisms, such as the up-regulation of other antioxidant enzymes and the secretion of exopolysaccharides. In an animal model of high-fat diet/low-dose streptozotocin-induced type 2 diabetes mellitus, LP strain L14 was associated with enhanced production of exopolysaccharides, microbial metabolites responsible for antioxidant properties [45,46,47]. In addition, in the genome annotation of the same LP strain, a system of antioxidant cellular components was found, comprised of thioredoxin, glutathione–glutaredoxin, superoxide dismutase, and glutathione peroxidase, suggesting that some LP strains could provide antioxidant characteristics [12].

The hepatic histological aspect of Dexa-treated rats displayed macrovesicular steatosis, hepatocyte degeneration, and inflammation surrounding the portal area, suggesting morphological alterations similar to those observed in NAFLD. These results, similar to those noticed in other animal studies, are related to the metabolic effects of glucocorticoids; steroids activate their specific receptors and consequently stimulate lipid deposition by increasing de novo lipogenesis and the uptake of free fatty acids released from adipocytes, leading to insulin resistance development [6,48]. The administration of SB probiotics in Dexa-treated rats reduced the accumulation of intracellular lipid droplets, but the hepatocyte degeneration and inflammatory infiltrates were not influenced. SB treatment decreased the lipid storage and inflammation within the liver and modulated the gut microbiota structure in an animal model of leptin-resistant obese and type 2 diabetic mice, suggesting that this dietary supplement yeast probiotic could impact the liver alteration by influencing the gut-liver axis [25]. The supplementation of SB lyo CNCM I-745 in an animal model of diet-induced non-alcoholic steatohepatitis had similar results, by suppressing the expression of inflammation and fibrogenic genes and increasing microbial diversity and gut integrity [33]. Moreover, the supplementation with LP probiotics in Dexa-treated rats led to the restoration of hepatic architecture, decreasing the lipid deposition within the liver cells and reducing the inflammatory infiltrate around portal areas. This observation suggests that LP probiotics are associated with improved liver histology in animal models of NAFLD. Treatment with a synbiotic containing the LP B21060 strain showed reduced inflammation and steatosis in the liver tissue in a rat model of feeding with a diet enriched in fat associated with reduced impairment of insulin signaling and preserved gut barrier integrity [49].

## 5. Conclusions

The current paper concludes that probiotics with SB or LP strain decrease serum glucose, modulate the levels of inflammatory cytokines, and reduce the hepatic accumulation of lipids induced by glucocorticoid administration. These results are similar to those previously reported by our lab and suggest that basal metabolism, gut microbiota, and systemic inflammation could be interconnected by pathways involving the intestinal, liver, and pancreatic tissue. In the current paper, we have shown that different probiotic administrations in glucocorticoid-treated rats have different metabolic results compared to those obtained by the administration of MSB regarding the metabolic and inflammatory profile [16]. However, the reduced sample of the study groups and the short administration of probiotics limits the applicability of probiotics administration at the beginning of Dexa exposure reflecting the need for randomized controlled trials.

## Figures and Tables

**Table 1:** The design of the experimental study.

**Group**	Negative control	Saline injection + CMC	Blood samples Liver removal
	2. Dexa	Dexa+ CMC	
	3. Dexa+ SB	Dexa+ SB probiotics	
	4. Dexa+ LP	Dexa+ LP probiotics	
	Day	1–7	8

Abbreviations: CMC, carboxymethylcellulose; Dexa, dexamethasone; SB, *Saccharomyces boulardii;* LP, *Lactobacillus paracasei*.

## Data Availability

The original contributions presented in this study are included in the article. Further inquiries can be directed to the corresponding author.

## Abbreviations

The following abbreviations are used in this manuscript:

CMC	Carboxymethylcellulose
CFU	Colony-forming units
Dexa	Dexamethasone
ELISA	Enzyme-linked immunosorbent assay
H&E	Hematoxylin-eosin
IL-10	Interleukin-10
i.p.	Intraperitoneal
LP	*Lactobacillus paracasei*
LPS	Lipopolysaccharides
NAFLD	Non-alcoholic fatty liver disease
NAS	Non-alcoholic Fatty Liver Disease Activity Score
TNF-α	Tumor Necrosis Factor-alpha
TAC	Total antioxidant capacity
SCFA	Short-chain fatty acids
SB	*Saccharomyces boulardii*
